# Effect of non-surgical periodontal treatment on glycemic control of patients with diabetes: a meta-analysis of randomized controlled trials

**DOI:** 10.1186/s13063-015-0810-2

**Published:** 2015-07-03

**Authors:** Quan Li, Sha Hao, Jie Fang, Jing Xie, Xiang-Hui Kong, Jian-Xin Yang

**Affiliations:** Center of Stomatology, the Second Affiliated Hospital of Soochow University, No.1055 Sanxiang Road, Soochow, 215004 P. R. China; Department of Integrated Chinese and Western Medicine, Union Hospital, Tongji Medical College, Huazhong University of Science and Technology, Wuhan, China; Department of Medical Oncology, JingmenTraditional Chinese Medicine Hospital, Jingmen, 448000 China; Department of Stomatology, the First Affiliated Hospital of Wenzhou Medical College, No.2 Fuxue Road, Wenzhou, China; Department of Cardiology, the People’s Hospital of Jingmen, Jingmen, 448000 China

**Keywords:** Chronic periodontitis, HbA1c, Diabetes mellitus type 2, Dental scaling, Root planning

## Abstract

**Objective:**

The present study aimed at investigating whether non-surgical periodontal treatment can reduce the Haemoglobin A1c (HbA1c) % level in type 2 diabetic patients.

**Methods:**

A search of the literature on English publications was performed in Cochrane Central, Medline, ISI Web of Knowledge and EMBASE (until 06 February 2014). An RCT was selected if the subject was type 2 diabetic patients diagnosed with chronic periodontitis, and compared HbA1c% change after non-surgical periodontal treatment alone for at least three months of the study duration. Weighted mean difference for pooled data and large sample size strata were calculated. Heterogeneity and publication bias were explored.

**Results:**

After the study selection process, only 9 RCTs were suitable. Compared to the control group, the pooled analysis (n=1082) showed −0.27 % (95 % CI:-0.46 % to −0.07 %, p= 0.007) absolute difference in HbA1c % with treatment while studies with sufficient sample size had HbA1c % change of −0.014 % (95 % CI:-0.18 % to 0.16 %, p= 0.87). Publication bias was marginally significant with Egger’s teat (p=0.045) but not with Begg’s test (p=0.72).

**Conclusion:**

The moderate reduction in HbA1c after the non-surgical therapy in patients with type 2 diabetes is consistent with previous systematic reviews. However, more large scale and high-quality RCTs are necessitated to confirm these results.

**Electronic supplementary material:**

The online version of this article (doi:10.1186/s13063-015-0810-2) contains supplementary material, which is available to authorized users.

## Introduction

Recently the relationship between diabetes mellitus and chronic periodontitis has attracted the attention of researchers worldwide. Many studies suggested a two-way relationship between diabetes mellitus and chronic periodontitis [1–5]. Poor glycemic control can induce the increased risk of periodontal disease or of its greater severity in patients with diabetes. Accordingly, DM2 (type 2 diabetes mellitus) is considered as one of the risk factors of periodontal disease [6, 7]. Enhanced glycemic control can alleviate the pathological progress of the periodontal disease [8, 9].

Periodontitis is diagnosed when there is gingival inflammation, periodontal ligament and alveolar bone loss, and apical migration of junctional epithelium [10, 11]. Clinical manifestations include increased probing pocket depth or clinical attachment loss. Therapies for periodontitis are composed of oral hygiene instruction, scaling, root planing, antibiotics, chlorhexidine, surgical treatment, or a combination of these [12].

The DM2 is regarded nowadays as a worldwide epidemic. According to the WHO(World Health Organization) the global upcoming estimative of the number of people with DM2 will reach 366 million in the year 2030 [13]. DM2 (a metabolic disease resulting from defects in insulin production, insulin activity, or both) can cause micro- and macrovascular complications. Glycemic control is crucial in preventing complications [14–17]. HbA1c represents serum glucose levels during the 120-day life of the red blood cell, and is a robust indicant of glycemic control [18].

Some previous meta-analyses observed a significant positive effect of non-surgical periodontal treatment on glycemic control despite lack of homogeneity and robustness among studies [19–22]. Since then, some new randomized controlled trials have been done. Therefore, it is time to provide an update to accumulate more evidence and evaluate the potential effect of non-surgical periodontal treatment on glycemic control.

## Review

### Methods

#### Search stratege

Four databases (Cochrane CENTRAL, ISI Web of Science, MEDLINE (via PubMed) and EMBASE) have been systematically searched using the search terms [see Additional file 1], from their inception to 01 April 2015. Moreover, a manual search was performed from the last 20 years of the following journals: Journal of Periodontology, Journal of Dental Research, Journal of Clinical Periodontology, Journal of Periodontal Research, Journal of Dentistry, Journal of the American Dental Association, Periodontology 2000, Journal of Clinical Dentistry, Clinical Oral Investigations, Clinical Diabetes, Diabetologia, Diabetes, Diabetes Care, and Diabetes Research and Clinical Practice. To be as comprehensive as possible, the reference lists of the searched publications were thoroughly cross-checked to avoid omission of relevant studies.

### Study selection criteria

The following inclusion criteria had to be met: (1) randomized controlled clinical trial (RCT); (2) population: type 2 diabetes mellitus diagnosed with periodontitis; (3) non-surgical periodontal treatment without adjunctive use of local drug delivery and systemic antibiotics at least 3 months of follow-up; (4) control group with no periodontal treatment or delayed treatment; (5) outcome: mean change in HbA1c level; and(6) only published studies in the English language were considered for inclusion.

**Fig. 1:**
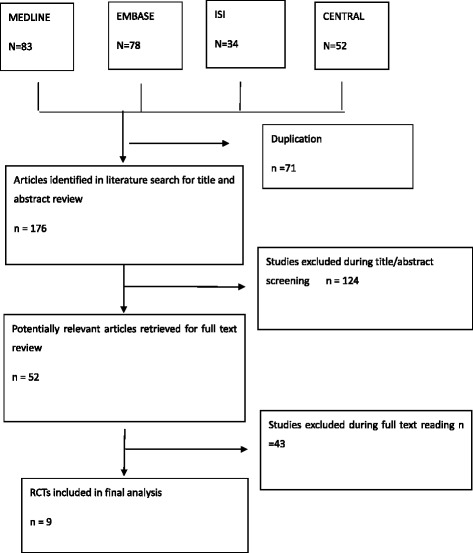
Flow Chart for the systematic review and meta-analysis

The Cochrane Handbook for Systematic Reviews of Interventions (Version 5.1.0) was used as a guideline for the selection process [23]. All retrieved articles were evaluated by four independent reviewers (QL, HS, JF and JXY). Disagreements were resolved by consensus. Figure 1 showed the reasons for exclusion. Since the databases were not mutually exclusive, reduplicative entries were removed.

After excluding articles ineligible for the study, the relevance of all articles were screened from the titles and abstracts. Full-text articles were assessed while articles which did not meet the pre-specified inclusion criteria were excluded.

### Methodological study quality assessment

Study quality was evaluated independently by two authors (HS and JF) with the following parameters, which were: (1) random sequence generation; (2) allocation concealment; (3) incomplete data outcome; (4) blinding; and (5) intention-to-treat analysis, as previously described [20].

### Data extraction

Four authors (QL, HS, JF and JXY) independently extracted data from each RCT in pre-designed forms. Any disagreements were resolved by consensus. From all relevant studies, the key parameters including demographics of the population, study quality, intervention in the control and treatment groups, follow-up duration and design, mean change of HbA1c levels in both groups were recorded. When not reported, the absolute difference of HbA1c level was calculated by extracting the mean change of HbA1c in the control group and the mean change of HbA1c in the intervention group. Standard deviation of the mean change of HbA1c level from baseline to follow-up was calculated according to a method previously described [24].

### Statistical analysis

All data for meta-analysis were submitted to STATA 12.0(Texas, USA). A meta-analysis was conducted, comparing the intervention group with non-surgical periodontal treatment and the control group which received no periodontal treatment or delayed treatment.

For a particular multi-arm study, the treatment group fulfilling the selection criteria for pair-wise comparison were included [23]. Thus, only the intervention group and control group contrasting non-surgical periodontal treatment alone versus no treatment/delayed treatment will be used for data analysis.

Weighted mean difference was calculated using a random effect model (Dersimonian-Laird method) from the included RCTs. In addition, the significant heterogeneity was assessed by chi-square test for homogeneity (Cochran Q-statistic and P-value), as well as I-squared statistic and 95 % CI of the I^2^. Publication bias was estimated by Begg’s test, Egger’s test and funnel plot.

## Results

The initial search from the four databases retrieved 247 articles, after removal of duplicates. The titles and abstracts of these studies were evaluated for inclusion, and the reasons for exclusion are showed in Fig. 1. Of these articles, only 52 articles were identified for full-text reading. Finally, a total of 9 RCTs were considered for the meta-analysis. No additional studies were identified by cross-checking the bibliographies of relevant and retrieved articles.

### Study characteristics

The characteristics of the 9 RCTs are listed in Table 1. All studies included were randomized controlled trials [25–33].The The mean age of subjects in the included studies ranged from 52.8 to 62.7 years. All selected studies are of subjects having both DM2 and chronic periodontitis. Scaling and root planning/curettage/debridement were regarded as the basic non-surgical periodontal treatment for the intervention group in all studies. In Singh et al., group B (n=15) was excluded for the meta-analysis because the adjunctive systemic doxycycline (100 mg daily for 14 days) was administrated [29].

**Table 1:** The characteristics of the included studies

Study	Year	Country	Number(n)				Interventions	Mean age years(SD)			Mean baseline			Follow-up	Study design and detail
								Male %			HbA1c % (SD)			time(months)	
			Total	C	Tx1	Tx2		C	Tx1	Tx2	C	Tx1	Tx2		
Kiran et al.	2005	Turkey	44	22	22		Control: delayed treatment Tx1: oral hygiene+SRP	52.82 (12.27) 36	55.95 (11.21) 45		7.0 (0.72)	7.31 (0.74)		3	RCT Single centre
															Blinding of assessor
Chen et al.	2012	China	134	44	45	45	Control: no treatment Tx1: SRP Tx2: supragingival prophylaxis	63.2 (8.51) 41.5	59.86 (9.48) 54.8	57.91 (11.35) 60.5	7.25 (1.49)	7.31 (1.23)	7.29 (1.55)	6	RCT Single centre
Koromantzos et al.	2012	Greece	60	30	30		Control:supragingival cleaning Tx:SRP in two sessions	59.42(9.8) 56.7	59.62(7.95) 53.3		7.59(0.66)	7.87(0.74)		3 and 6 months	RCT Single centre
															Blinding of assessor
Moeintaghavi et al.	2012	Iran	40	18	22		Control: delayed treatment Tx1: SRP	53.5(9.51) 61.1	56.27(10.52) 40.9	Overall 50.29(3)	8.72 (2.22)	8.15 (1.18)		3	RCT Single centre Blinding of assessor
Singh et al.	2008	India	45	15	15	15	Control:no treatment Tx1:SRP Tx2: SRP+systemic doxycycline	59.3(10.8) 56.3	52.8(10.36) 48.8		8.08(0.78)	7.93(0.78)	8.33(0.72)	3	RCT Single centre
Zhang et al.	2013	China	71	22	49		Control:no treatment Tx:SRP	62.7(10.7) 42.9	60.4(9.77) 45.5		7.38(1.30)	7.68(1.22)		3 and 6 months	RCT Single centre Blinding of assessor
Engebretson et al.	2013	USA	514	257	257		Control:no treatment Tx:SRP+mouthwash	57.9(9.6) 42.1	56.7(10.5) 45.6		7.78(0.60)	7.84(0.65)		3 and 6 months	RCT Multicenter Blinding of assessor
Gay IC et al.	2014	USA	126	66	60		Control:oral hygiene instruction Tx1: scaling +SRP	41.7(10.2) 58.3	45.5(9.0) 54.6		8.4(2.0)	9.0(2.3)		3	RCT Single centre
Raman RP et al.	2014	Malaysia	32	17	15		Control:oral hygiene instruction Tx1: scaling +SRP	73.3( 9.9) 26.7	52.9(6.2) 47.1		7.6 (1.5)	7.8 (1.5)		3	RCT Single centre

### Methodological study quality assessment

Risk of bias was assessed for each selected study by the following parameters, which are: random sequence generation, allocation concealment, blinding of participants and personnel, incomplete data outcome and intention-to-treat analysis. (Table 2). Only Engebretson et al. and Koromantzos et al. reported implementing intention-to-treat analysis [27, 31]. Allocation concealment was not observed in 3 studies [25–27].

**Table 2:** Quality measure of included studies in the meta-analysis

	Random sequence generation	Allocation concealment	Blinding of participants and personnal	Incomplete outcome data addressed	Intention-to-treat analysis	Selecting reporting
Kiran 2005	?	?	?	+	?	+
Chen 2012	+	?	+	+	-	+
Moeintaghavi 2012	+	?	?	+	?	+
Koromantzos 2012	+	+	+	+	+	+
Singh 2008	?	-	+	+	?	+
Zhang 2013	+	+	+	+	-	+
Engebretson 2013	+	+	+	+	+	+
Gay IC 2014	+	+	+	+	+	+
Raman RP 2014	+	?	?	+	?	+

+ adequate, ? unclear, − inadequate

### Pooled analysis

**Fig. 2:**
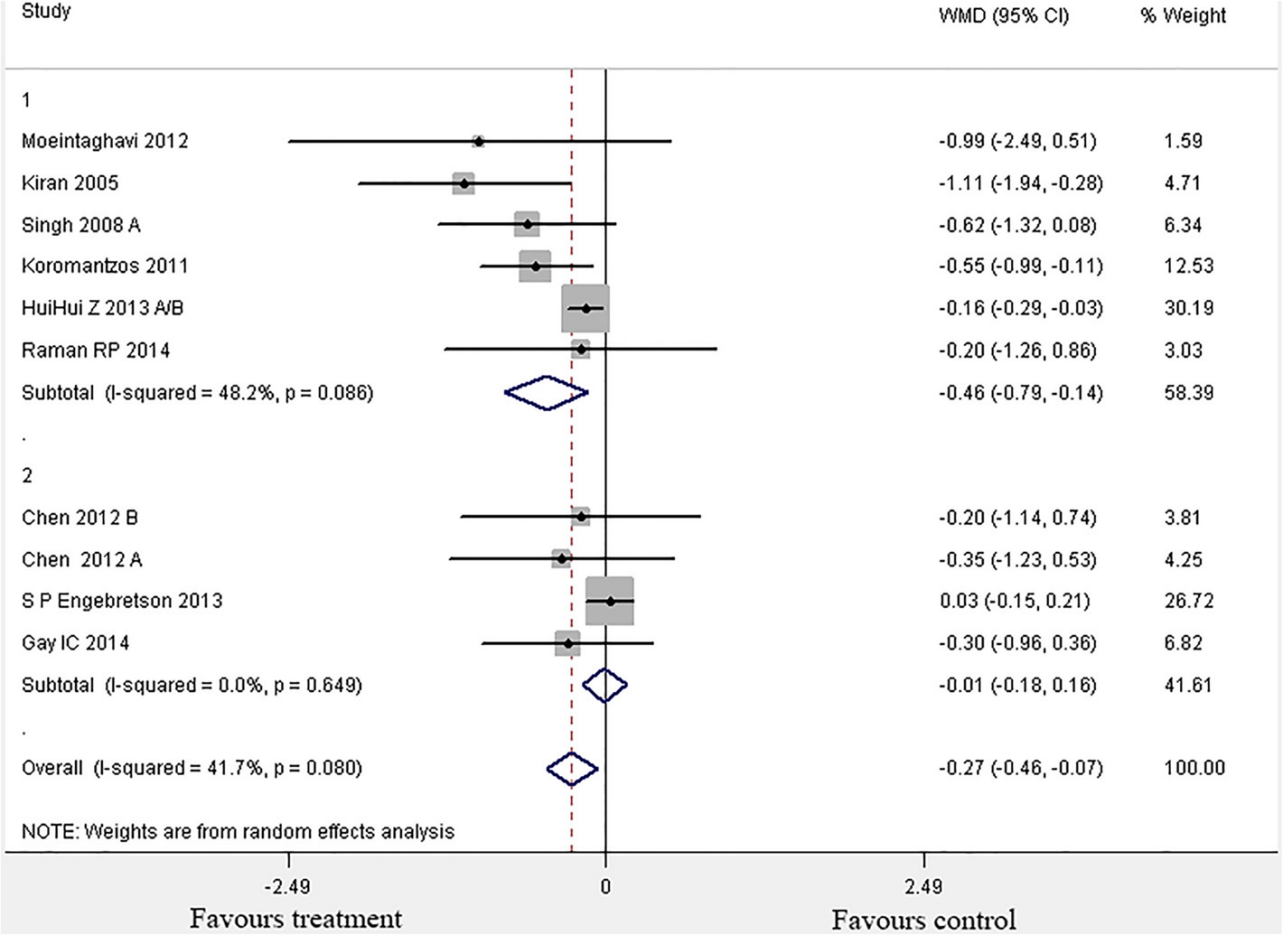
Forest plot of comparison: Periodontal Therapy outcome: Change in HbA1c (%HbA1c) at 3 months

A statistically significant difference in HbA1c reduction was seen in the pooled analysis (Fig. 2) between the intervention and control groups (p= 0.007), with an effect size of −0.27 % (95 % CI:-0.46 % to −0.07 %).

### Exploration of heterogeneity

The heterogeneity (I^2^) among the 9 studies was 41.7 % (X^2^ =15.43, p=0.080), which suggested moderate heterogeneity.

Since sample size was identified as a potential source of heterogeneity, then subgroup analysis was conducted. Non-surgical periodontal intervention with small sample size (n<80) showed a significant improvement (p=0.005) of HbA1c level [−0.46 % (95 % CI:-0.79 % to −0.14 %)]. Also, the heterogeneity among studies was moderate (x^2^= 9.64,p=0.086, I^2^=48.2 %)(25,27-30). However, this improvement was not significant (p=0.65) with large sample size trials, with HbA1c % change of 0.01 % (95 % CI:-0.18 % to 0.16 %, p= 0.87) [26, 31, 32].

### Publication bias

**Fig. 3:**
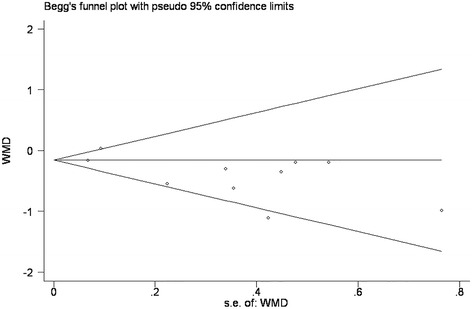
Funnel plot of comparison: periodontal therapy outcome: change in HbA1c

Visual inspection of the funnel plot revealed an asymmetrical distribution (Fig. 3). In Egger’s test, the intercept value of the y-axis was negative (−2.37). The publication bias was marginally significant in Egger’s test (p=0.045), therefore caution should be taken to get the conclusion of no small-study effects. However, Begg’s test was not statistically significant (p=0.72).

## Discussion

This review updates a previous systematic review [19] that was published in 2013, due to an increase of some new large-sample RCTs in this subject. This one contains all previous RCTs [25–29] in that review, and new studies meeting the inclusion criteria have been added. In all, nine randomized clinical trials [25–33] are retrieved, with two new studies [30, 31] meet the selection criteria established by Sgolastra et al. [19]. Now the total number of the population of this meta-analysis amount to 1082, nearly three times the subjects of the meta-analysis by Sgolastra et al. [19]. It is noteworthy that the standard deviation of the HbA1c reduction ranges in the present analysis from 0.12 to 2.87, which is consistent with previous reviews [19–22].

The moderate effect size of −0.27 % HbA1c (95 % CI:-0.46,-0.07) observed among all nine studies remains statistically significant, which is consistent with the two recent systematic reviews [−0.36 % HbA1c,CI:-0.54,-0.19][21]and [−0.65 % HbA1c, CI:-0.43,-0.88] [19]. Therefore, the present meta-analysis seemingly reaches the conclusion that non-surgical periodontal intervention may reduce the HbA1c level [19]. However, caution should be taken to interpret the data as the heterogeneity among studies was margionally significant (p=0.080).

When stratified by sample size (Fig. 2), the analysis showed that trials with small sample size (n<80) demonstrated effective improvement of glycemic control, reducing 0.46 % HbA1c level compared with no treatment [25, 27–30, 33]. The subgroup analysis of small RCTs showed a greater effect size, partially due to the small sample size effect(the small sample size study with larger variance is more likely to overestimate the effect sizes compared with large trials).

Conversely RCTs with large sample size (n>80) showed an insignificant effect size [26, 31, 32]. While recent studies suggested that TNF-a enhanced the insulin resistance in infection by inhibiting insulin-induced tyrosine phosphorylation of insulin receptor substrate-1(IRS-1) [34]. Biological plausibility could be established at this point. Meanwhile only three trials adopted the large sample size within the study design [26, 31, 32]. Therefore, more large-multicenter high-quality RCTs are needed in this area.

Furthermore, the methodological study quality was far from ideal. For instance, per protocol analysis was done in Chen et al. [26], Moeintaghavi et al. [28] and Zhang et al. [30]. This could jeopardize the generation of random sequence. The duration of treatment ranged from three to six months. Although all studies provided mechanical instrumentation as non-surgical periodontal treatment, Engebretson et al. [31] provided chlorhexidine oral rinse at baseline and supportive periodontal therapy at 3 and 6 months. Chen et al. [26] prescribed only supragingival prophylaxis at 3 months in the treatment group 3 with no debridement in deep periodontal pockets. Discrepancies in baseline HbA1c levels, treat regimens of DM2 and the severity of CP could also account to some extent for the heterogeneity.

The moderate effect size of the reduction of HbA1c −0.27 % HbA1c (95 % CI:-0.46,-0.07) was observed among all nine studies. While the clinical value of the HbAbc-reduction can not be neglected. Non-surgical periodontal therapy may be an adjunctive way to enhance glycemic control [34]. It has been suggested that 1 % reduction in HbA1c levels means 35 % decrease in complications. It also estimated that a 10 % decrease in mortality is associated with a 0.2 % reduction in HbA1c [35]. Therefore, despite the modest effect size of the reduction of HbA1c level, non-surgical periodontal treatment regimens enhances the glycemic control.

The precise pathogenesis involving CP and DM2 needs to be further investigated. Chronic periodontitis may initiate or maintain an elevated systemic chronic inflammation. Enhanced serum level of TNF-a, IL-6 and IL-1 can be detected in patients diagnosed with CP and DM2, IL-1 and IL-6 can antagonize the insulin action and TNF-a can interfere with lipid metabolism [36]. Currently several studies have demonstrated that IL-1 antagonism [37–39] and salsalate [40] may get the improved glycemic control of the DM2 with CP.

The strengths of this meta-analysis are the restriction to non-surgical periodontal treatment alone, without the adjunctive antibiotics which could interfere the mean change of HbA1c, and the inclusion of the largest multicenter high-quality RCT [31] since the publication of the four reviews in 2013. The outcome suggested that more large sample size and high-quality RCTs are needed to confirm the conclusion.

However, some limitations in the present study should be recognized. The small sample size within the study could lead to the small-study effect. Furthermore, discrepancies in baseline HbA1c levels, treat regimens of DM2 and CP severity were observed among the included studies.

In order to clarify the biological mechanism of the effect of SRP on the change of HbA1c level, it is important that negative findings in this area should be objectively published.

## Conclusion

The meta-analysis result seems to support the effectiveness of non-surgical periodontal treatment in the enhancement of glycemic control in patients with CP and DM2. However, more large-multicenter high-quality RCTs are needed to confirm these results.

## Additional file

Additional file 1:
**Search strategies in Cochrane central, Pubmed, Embase.**

## Abbreviations

HbA1c: Haemoglobin A1c
DM2: Type 2 diabetes mellitus
WHO: World Health Organization
RCT: Randomized clinical trials
MD: Mean difference
SE: Standard error
