# Supplementary material for: Effect of non-surgical periodontal treatment on glycemic control of patients with diabetes: a meta-analysis of randomized controlled trials
Source: Trials. 2015 Jul 3;16:291. doi: 10.1186/s13063-015-0810-2 (PMC4490675; doi:10.1186/s13063-015-0810-2)
Supplement: Additional file 1: — Search strategies in Cochrane central, Pubmed, Embase. [file 13063_2015_810_MOESM1_ESM.doc]

**Additional file 1. Search strategies**

**Cochrane central, Pubmed, Embase.** **WEB OF SCIENCE**

CENTRAL search strategy

| #1 | [MeSH descriptor DENTAL PROPHYLAXIS explode all trees](http://onlinelibrary.wiley.com.ezp-prod1.hul.harvard.edu/o/cochrane/searchHistory?mode=runquery&qnum=1) |
| --- | --- |
| #2 | [MeSH descriptor DENTAL SCALING explode all trees](http://onlinelibrary.wiley.com.ezp-prod1.hul.harvard.edu/o/cochrane/searchHistory?mode=runquery&qnum=2) |
| #3 | [MeSH descriptor ROOT PLANING explode all trees](http://onlinelibrary.wiley.com.ezp-prod1.hul.harvard.edu/o/cochrane/searchHistory?mode=runquery&qnum=3) |
| #4 | [MeSH descriptor PERIODONTAL DEBRIDEMENT explode all trees](http://onlinelibrary.wiley.com.ezp-prod1.hul.harvard.edu/o/cochrane/searchHistory?mode=runquery&qnum=4) |
| #5 | [MeSH descriptor SUBGINGIVAL CURETTAGE explode all trees](http://onlinelibrary.wiley.com.ezp-prod1.hul.harvard.edu/o/cochrane/searchHistory?mode=runquery&qnum=5) |
| #6 | (dental:ab,ti) OR (subgingival:ab,ti) OR (supragingival:ab,ti) OR (periodontal:ab,ti) OR (pocket:ab,ti) OR (ultrasonic:ab,ti) OR (mechanical:ab,ti) |
| #7 | [(scaling*:ab,ti) OR (planing*:ab,ti) OR (debridement*:ab,ti) OR (curettage*:ab,ti) OR (irrigation*:ab,ti) OR (instrumentation*:ab,ti)](http://onlinelibrary.wiley.com.ezp-prod1.hul.harvard.edu/o/cochrane/searchHistory?mode=runquery&qnum=7) |
| #8 | [(#6 AND #7)](http://onlinelibrary.wiley.com.ezp-prod1.hul.harvard.edu/o/cochrane/searchHistory?mode=runquery&qnum=8) |
| #9 | [("dental prophylaxis":ab,ti)](http://onlinelibrary.wiley.com.ezp-prod1.hul.harvard.edu/o/cochrane/searchHistory?mode=runquery&qnum=9) |
| #10 | [("periodontal therapy":ab,ti)](http://onlinelibrary.wiley.com.ezp-prod1.hul.harvard.edu/o/cochrane/searchHistory?mode=runquery&qnum=10) |
| #11 | [("periodontal therapeutics":ab,ti)](http://onlinelibrary.wiley.com.ezp-prod1.hul.harvard.edu/o/cochrane/searchHistory?mode=runquery&qnum=11) |
| #12 | [("periodontal treatment":ab,ti)](http://onlinelibrary.wiley.com.ezp-prod1.hul.harvard.edu/o/cochrane/searchHistory?mode=runquery&qnum=12) |
| #13 | [("periodontal intervention":ab,ti)](http://onlinelibrary.wiley.com.ezp-prod1.hul.harvard.edu/o/cochrane/searchHistory?mode=runquery&qnum=13) |
| #14 | [("periodontal interventions":ab,ti)](http://onlinelibrary.wiley.com.ezp-prod1.hul.harvard.edu/o/cochrane/searchHistory?mode=runquery&qnum=14) |
| #15 | [("periodontal maintenance":ab,ti)](http://onlinelibrary.wiley.com.ezp-prod1.hul.harvard.edu/o/cochrane/searchHistory?mode=runquery&qnum=15) |
| #16 | [(#1 OR #2 OR #3 OR #4 OR #5 OR #8 OR #9 OR #10 OR #11 OR #12 OR #13 OR #14 OR #15)](http://onlinelibrary.wiley.com.ezp-prod1.hul.harvard.edu/o/cochrane/searchHistory?mode=runquery&qnum=16) |
| #17 | [MeSH descriptor DIABETES MELLITUS explode all trees](http://onlinelibrary.wiley.com.ezp-prod1.hul.harvard.edu/o/cochrane/searchHistory?mode=runquery&qnum=17) |
| #18 | [MeSH descriptor HEMOGLOBIN A NEAR/ GLYCOSYLATED explode all trees](http://onlinelibrary.wiley.com.ezp-prod1.hul.harvard.edu/o/cochrane/searchHistory?mode=runquery&qnum=18) |
| #19 | [("hb a1a b":ab,ti)](http://onlinelibrary.wiley.com.ezp-prod1.hul.harvard.edu/o/cochrane/searchHistory?mode=runquery&qnum=19) |
| #20 | [("hb a1c":ab,ti)](http://onlinelibrary.wiley.com.ezp-prod1.hul.harvard.edu/o/cochrane/searchHistory?mode=runquery&qnum=20) |
| #21 | [(hba1:ab,ti)](http://onlinelibrary.wiley.com.ezp-prod1.hul.harvard.edu/o/cochrane/searchHistory?mode=runquery&qnum=21) |
| #22 | [("glycosylated hemoglobin":ab,ti)](http://onlinelibrary.wiley.com.ezp-prod1.hul.harvard.edu/o/cochrane/searchHistory?mode=runquery&qnum=22) |
| #23 | [("hb a1":ab,ti)](http://onlinelibrary.wiley.com.ezp-prod1.hul.harvard.edu/o/cochrane/searchHistory?mode=runquery&qnum=23) |
| #24 | [("glycosylated a1b":ab,ti)](http://onlinelibrary.wiley.com.ezp-prod1.hul.harvard.edu/o/cochrane/searchHistory?mode=runquery&qnum=24) |
| #25 | [("hb a1b":ab,ti)](http://onlinelibrary.wiley.com.ezp-prod1.hul.harvard.edu/o/cochrane/searchHistory?mode=runquery&qnum=25) |
| #26 | [("hb a1a":ab,ti)](http://onlinelibrary.wiley.com.ezp-prod1.hul.harvard.edu/o/cochrane/searchHistory?mode=runquery&qnum=26) |
| #27 | [("glycated hemoglobin":ab,ti)](http://onlinelibrary.wiley.com.ezp-prod1.hul.harvard.edu/o/cochrane/searchHistory?mode=runquery&qnum=27) |
| #28 | [("glycated hemoglobins":ab,ti)](http://onlinelibrary.wiley.com.ezp-prod1.hul.harvard.edu/o/cochrane/searchHistory?mode=runquery&qnum=28) |
| #29 | [("glycosylated haemoglobins":ab,ti)](http://onlinelibrary.wiley.com.ezp-prod1.hul.harvard.edu/o/cochrane/searchHistory?mode=runquery&qnum=29) |
| #30 | [("glycosylated haemoglobin":ab,ti)](http://onlinelibrary.wiley.com.ezp-prod1.hul.harvard.edu/o/cochrane/searchHistory?mode=runquery&qnum=30) |
| #31 | [("glycated haemoglobin":ab,ti)](http://onlinelibrary.wiley.com.ezp-prod1.hul.harvard.edu/o/cochrane/searchHistory?mode=runquery&qnum=31) |
| #32 | [("glycated haemoglobins":ab,ti)](http://onlinelibrary.wiley.com.ezp-prod1.hul.harvard.edu/o/cochrane/searchHistory?mode=runquery&qnum=32) |
| #33 | [("glycemic control":ab,ti)](http://onlinelibrary.wiley.com.ezp-prod1.hul.harvard.edu/o/cochrane/searchHistory?mode=runquery&qnum=33) |
| #34 | [("glycaemic control":ab,ti)](http://onlinelibrary.wiley.com.ezp-prod1.hul.harvard.edu/o/cochrane/searchHistory?mode=runquery&qnum=34) |
| #35 | [(diabetes:ab,ti)](http://onlinelibrary.wiley.com.ezp-prod1.hul.harvard.edu/o/cochrane/searchHistory?mode=runquery&qnum=35) |
| #36 | [(#17 OR #18 OR #19 OR #20 OR #21 OR #22 OR #23 OR #24 OR #25 OR #26 OR #27 OR #28 OR #29 OR #30 OR #31 OR #32 OR #33 OR #34 OR #35)](http://onlinelibrary.wiley.com.ezp-prod1.hul.harvard.edu/o/cochrane/searchHistory?mode=runquery&qnum=36) |
| #37 | [(#16 AND #36)](http://onlinelibrary.wiley.com.ezp-prod1.hul.harvard.edu/o/cochrane/searchHistory?mode=runquery&qnum=37) |
| #38 | [(#37)](http://onlinelibrary.wiley.com.ezp-prod1.hul.harvard.edu/o/cochrane/searchHistory?mode=runquery&qnum=38) in Trials |
|  | 9-15 "dental prophylaxis":ab,ti or "periodontal therapy":ab,ti or "periodontal therapeutics":ab,ti or "periodontal treatment":ab,ti or "periodontal intervention":ab,ti or "periodontal interventions":ab,ti or "periodontal maintenance":ab,ti  19-35 "hb a1a b":ab,ti or "hb a1c":ab,ti or hba1:ab,ti or "glycosylated hemoglobin":ab,ti or "hb a1":ab,ti or "glycosylated a1b":ab,ti or "hb a1b":ab,ti or "hb a1a":ab,ti or "glycated hemoglobin":ab,ti or "glycated hemoglobins":ab,ti or "glycosylated haemoglobins":ab,ti or "glycosylated haemoglobin":ab,ti or "glycated haemoglobin":ab,ti or "glycated haemoglobins":ab,ti or "glycemic control":ab,ti or "glycaemic control":ab,ti or diabetes:ab,ti |

MEDLINE search strategy

(Dental Prophylaxis[Mesh] OR Dental Scaling[Mesh] OR Root Planing[Mesh] OR Periodontal Debridement[Mesh] OR Subgingival Curettage[Mesh] OR ((dental[tiab] OR subgingival[tiab] OR supragingival[tiab] OR periodontal[tiab] OR pocket[tiab] OR ultrasonic[tiab] OR mechanical[tiab])

AND (scaling*[tiab] OR planing*[tiab] OR debridement*[tiab] OR curettage*[tiab] OR irrigation*[tiab] OR instrumentation*[tiab])) OR dental prophylaxis[tiab] OR periodontal therapy[tiab] OR periodontal therapeutics[tiab] OR periodontal treatment[tiab] OR periodontal intervention*[tiab] OR periodontal maintenance[tiab])

AND (Diabetes Mellitus[Mesh] OR Hemoglobin A, Glycosylated[Mesh] OR hemoglobin A1c protein, human[Supplementary Concept] OR pre-hemoglobin A, glycosylated[Supplementary Concept] OR Hb A1a+b[tiab] OR Hb A1c[tiab] OR Hb A1[tiab] OR glycosylated hemoglobin*[tiab] OR Hb A1[tiab] OR glycosylated A1b[tiab] OR Hb A1b[tiab] OR Hb A1a[tiab] OR glycated hemoglobin*[tiab] OR glycosylated haemoglobin*[tiab] OR glycated haemoglobin*[tiab] OR glycemic control[tiab] OR glycaemic control[tiab] OR diabetes[tiab])

AND randomized controlled trials

EMBASE search strategy

'periodontics'/de OR ((dental:ab,ti OR subgingival:ab,ti OR supragingival:ab,ti OR periodontal:ab,ti OR pocket:ab,ti OR ultrasonic:ab,ti OR mechanical:ab,ti) AND (scaling*:ab,ti OR planing*:ab,ti OR debridement*:ab,ti OR curettage*:ab,ti OR irrigation*:ab,ti OR instrumentation*:ab,ti)) OR 'dental prophylaxis':ab,ti OR 'periodontal therapy':ab,ti OR 'periodontal therapeutics':ab,ti OR 'periodontal treatment':ab,ti OR 'periodontal intervention':ab,ti OR 'periodontal interventions':ab,ti OR 'periodontal maintenance':ab,ti

AND ('glycosylated hemoglobin'/exp OR 'hb a1a+b':ab,ti OR 'hb a1c':ab,ti OR hba1:ab,ti OR 'glycosylated hemoglobin':ab,ti OR 'hb a1':ab,ti OR 'glycosylated a1b':ab,ti OR 'hb a1b':ab,ti OR 'hb a1a':ab,ti OR 'glycated hemoglobin':ab,ti OR 'glycated hemoglobins':ab,ti OR 'glycosylated haemoglobins':ab,ti OR 'glycosylated haemoglobin':ab,ti OR 'glycated haemoglobin':ab,ti OR 'glycated haemoglobins':ab,ti OR 'glycemic control':ab,ti OR 'glycaemic control':ab,ti OR diabetes:ab,ti)

AND random*:ab,ti OR 'randomized controlled trial':ab,ti or 'randomized controlled trial'/exp or 'randomized controlled trial (topic)'/exp
